# Hyperactivated mTORC1 downregulation of FOXO3a/PDGFRα/AKT cascade restrains tuberous sclerosis complex-associated tumor development

**DOI:** 10.18632/oncotarget.18963

**Published:** 2017-07-04

**Authors:** Li Wang, Zhaofei Ni, Yujie Liu, Shuang Ji, Fuquan Jin, Keguo Jiang, Junfang Ma, Cuiping Ren, Hongbing Zhang, Zhongdong Hu, Xiaojun Zha

**Affiliations:** ^1^ Department of Biochemistry and Molecular Biology, School of Basic Medicine, Anhui Medical University, Hefei, China; ^2^ The First Clinical Medical School, Anhui Medical University, Hefei, China; ^3^ Department of Nephrology, The Third Affiliated Hospital of Anhui Medical University, Hefei, China; ^4^ Department of Neurology, Beijing Shijitan Hospital, Capital Medical University, Beijing, China; ^5^ Department of Parasitology, School of Basic Medicine, Anhui Medical University, Hefei, China; ^6^ State Key Laboratory of Medical Molecular Biology, Department of Physiology and Pathophysiology, Institute of Basic Medical Sciences, Chinese Academy of Medical Sciences and Peking Union Medical College, Beijing, China; ^7^ Modern Research Center for Traditional Chinese Medicine, School of Chinese Materia Medica, Beijing University of Chinese Medicine, Beijing, China

**Keywords:** mTOR, FOXO3a, PDGFRα, AKT, tumorigenesis

## Abstract

Hyperactivation of mammalian target of rapamycin complex 1 (mTORC1), caused by loss-of-function mutations in either the TSC1 or TSC2 gene, leads to the development of tuberous sclerosis complex (TSC), a benign tumor syndrome with multiple affected organs. mTORC1-mediated inhibition of AKT constrains the tumor progression of TSC, but the exact mechanisms remain unclear. Herein we showed that loss of TSC1 or TSC2 downregulation of platelet-derived growth factor receptor α (PDGFRα) expression was mediated by mTORC1. Moreover, mTORC1 inhibited PDGFRα expression via suppression of forkhead box O3a (FOXO3a)-mediated PDGFRα gene transcription. In addition, ectopic expression of PDGFRα promoted AKT activation and enhanced proliferation and tumorigenic capacity of Tsc1- or Tsc2-null mouse embryonic fibroblasts (MEFs), and vice versa. Most importantly, rapamycin in combination with AG1295, a PDGFR inhibitor, significantly inhibited growth of TSC1/TSC2 complex-deficient cells *in vitro* and *in vivo*. Therefore, downregulated FOXO3a/PDGFRα/AKT pathway exerts a protective effect against hyperactivated mTORC1-induced tumorigenesis caused by loss of TSC1/TSC2 complex, and the combination of rapamycin and AG1295 may be a new effective strategy for TSC-associated tumors treatment.

## INTRODUCTION

The mammalian target of rapamycin (mTOR) is a conserved serine/threonine protein kinase implicated in a wide array of cellular processes including cell proliferation, growth, metabolism, and autophagy [1–4]. mTOR forms at least two distinct multi-protein complexes, mTOR complex 1 (mTORC1) and mTOR complex 2 (mTORC2). mTORC1 is composed of mTOR, Raptor, mLST8, PRAS40, and Deptor, which is inhibited by rapamycin and its analogs, whereas mTORC2 consists of mTOR, Rictor, and several other binding proteins and is rapamycin-insensitive [5–7]. mTORC1 integrates a wide range of upstream signals from growth factors, nutrients, oxygen, and energy status to orchestrate cell growth, metabolism, and survival [1, 8].

Tuberous sclerosis complex (TSC) is an autosomal dominant disorder characterized by the formation of benign tumors in multiple organs including kidney, brain, skin, and heart, which is caused by inactivating mutations in either of two tumor suppressor genes: *TSC1* or *TSC2* [9–11]. TSC1 and TSC2 form a functional complex that is a suppressor upstream of mTORC1 [12, 13]. Loss-of-function mutations in either the *TSC1* or *TSC2* gene lead to activation of mTORC1, which is believed to be responsible for the tumor development in TSC [9, 14–16]. Interestingly, patients with TSC rarely develop malignant lesions. It has been reported that mTORC1-mediated negative feedback inhibition of AKT contributes to the benign nature of TSC tumors [17–20]. However, the underlying mechanisms remain poorly understood.

Platelet-derived growth factor (PDGF) stimulates cell proliferation, survival, angiogenesis, and migration via binding to α and β tyrosine kinase receptors, PDGFRα and PDGFRβ [21, 22]. It has been reported that PDGF-related signaling is linked to the pathogenesis of fibrotic disorders, atherosclerosis, and cancers [18, 23]. Intriguingly, PDGFRα and PDGFRβ show distinct effects in the development of human diseases [24–26]. We have shown that loss of TSC1/TSC2 complex reduced the expression of PDGFRα and PDGFRβ [17], and restoration of PDGFRβ enhanced tumorigenic potential of TSC1/TSC2 complex-deficient cells [18]. However, the function and regulation of PDGFRα in TSC-associated tumors remain elusive.

In this study, we demonstrated that hyperactivated mTORC1 downregulated PDGFRα expression through inhibition of forkhead box O3a (FOXO3a), which led to inactivation of AKT and subsequently attenuated the tumorigenicity of Tsc1- or Tsc2-null mouse embryonic fibroblasts (MEFs). In addition, the combination of rapamycin and AG1295, a specific PDGFR inhibitor, may be exploited as a novel regimen for the treatment of TSC-related cancers.

## RESULTS

### Loss of TSC1 or TSC2 reduced PDGFRα expression through activation of mTORC1

**Figure 1:**
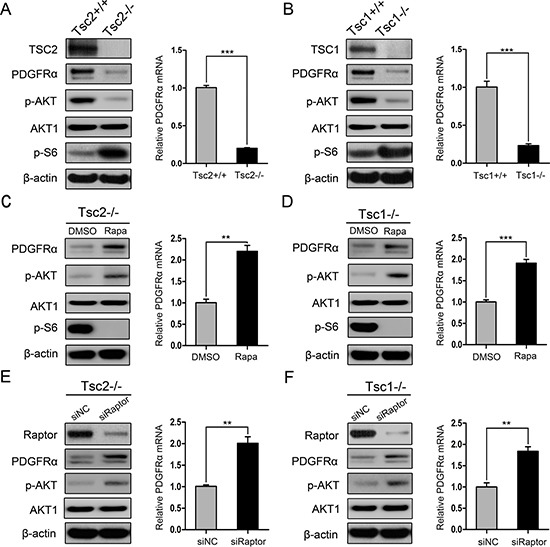
mTORC1 is a negative regulator of PDGFRα (**A**) Tsc2+/+ and Tsc2−/− MEFs. (**B**) Tsc1+/+ and Tsc1−/− MEFs. (**C**, **D**) Tsc2−/− (C) or Tsc1−/− MEFs (D) were treated with DMSO or with 20 nM rapamycin (Rapa) for 24 h. (**E**, **F**) Tsc2−/− (E) or Tsc1−/− MEFs (F) were transfected with the control siRNAs (siNC) or siRNAs targeting Raptor for 48 h. A–F. Cell lysates were subjected to immunoblotting with the indicated antibodies (left panels). qRT-PCR was performed to detect the mRNA level of PDGFRɑ (right panels). **P < 0.01; ***P < 0.001.

Consistent with previous studies [17, 18], loss of TSC1 or TSC2 induced mTORC1 activation (p-S6 is an indicator of mTORC1 activity) and AKT inhibition (Figure 1A and 1B). Also, loss of TSC1 or TSC2 led to decreased expression of PDGFRα at both protein and mRNA levels (Figure 1A and 1B). To investigate whether mTORC1 is involved in the regulation of PDGFRα expression, we first examined the effect of rapamycin, a specific mTORC1 inhibitor, on PDGFRα expression. As shown in Figure 1C and 1D, rapamycin treatment markedly increased PDGFRα expression in Tsc2−/− or Tsc1−/− MEFs. To further verify that the negative regulation of PDGFRα by loss of TSC1/TSC2 complex is mediated by mTORC1, we examined PDGFRα levels in Tsc2−/− or Tsc1−/− MEFs with knockdown of Raptor (a specific component of mTORC1). As shown in Figure 1E and 1F, depletion of Raptor with siRNAs increased PDGFRα levels in Tsc2- or Tsc1-null MEFs. However, knockdown of Rictor (a specific component of mTORC2) had a minimal effect on the expression of PDGFRα in Tsc2- or Tsc1-null MEFs (Supplementary Figure 1). Taken together, hyperactivated mTORC1 is responsible for the downregulation of PDGFRα due to loss of TSC1/TSC2 complex.

### Decreased PDGFRα blunts the proliferation and tumorigenesis of Tsc1- or Tsc2-deficient cells

**Figure 2:**
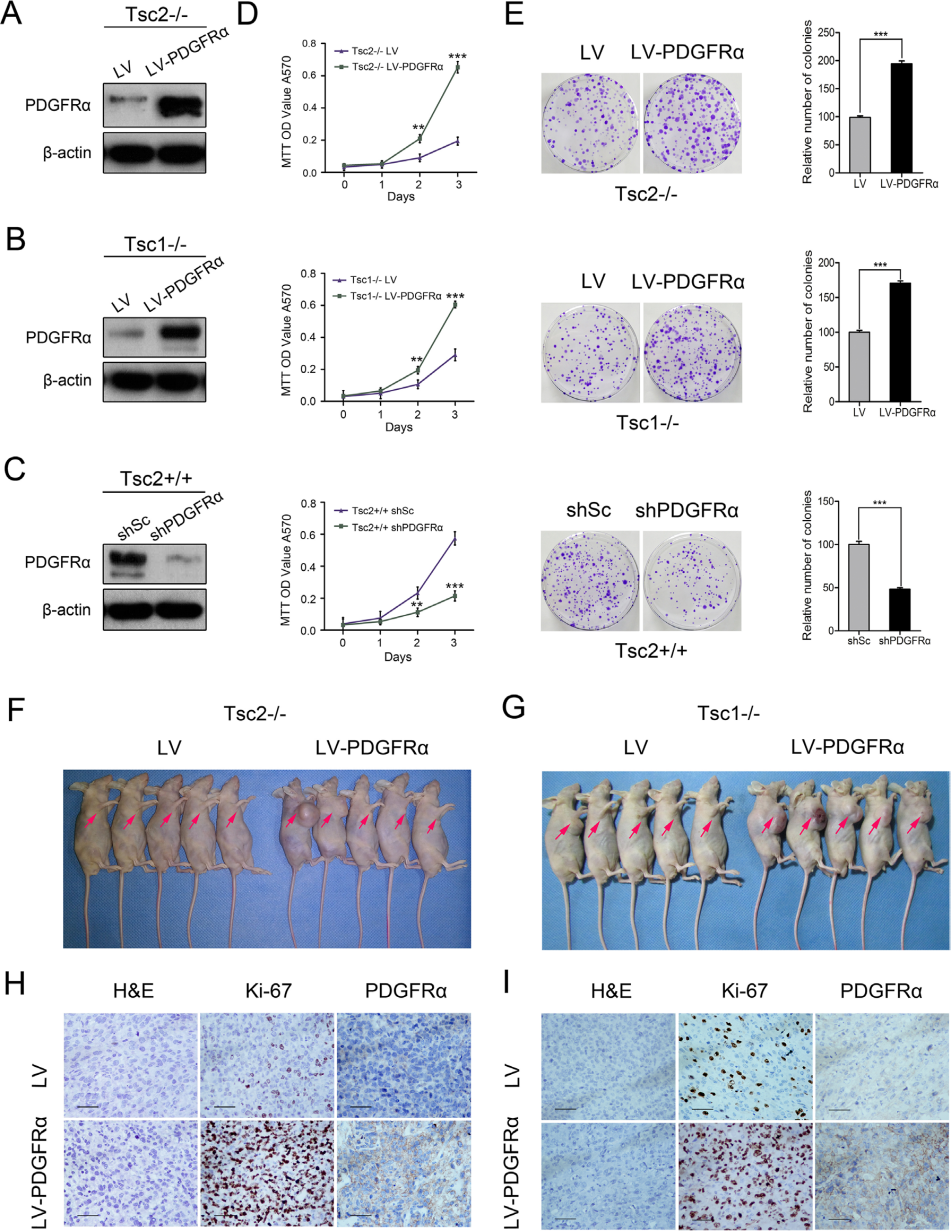
Decreased PDGFRα inhibits the growth of cells with deficiency of TSC1/TSC2 complex *in vitro* and *in vivo* (**A**, **B**) Tsc2−/− (A) or Tsc1−/− MEFs (B) were infected with lentivirus harboring a vector encoding PDGFRɑ (LV-PDGFRɑ) or the empty vector (LV). (**C**) Tsc2+/+ MEFs stably expressing shRNAs targeting PDGFRɑ (shPDGFRɑ) or a control shRNA (shSc). A–C. Cell lysates were subjected to immunoblotting with the indicated antibodies. (**D**) Proliferation of the indicated cells was examined using an MTT assay. (**E**) The colonies formed by the indicated cells were stained and counted. Representative images (left panels) and quantifications (right panels). (**F**, **G**) Tsc2−/− (F) or Tsc1−/− MEFs (G) transduced with LV-PDGFRɑ or LV lentiviruses were inoculated subcutaneously into nude mice, followed by monitoring for tumor growth. (**H**, **I**) Tumor tissues derived from Tsc2−/− (H) or Tsc1−/− MEFs (I) were fixed and embedded with paraffin, and then subjected to H&E and immunohistochemical staining. Representative images were presented. Scale bar, 50 μm. **P < 0.01; ***P < 0.001.

To evaluate the role of PDGFRα in TSC tumors, Tsc2-null MEFs were infected with lentiviruses for overexpressing PDGFRα, which was confirmed by western blot analysis (Figure 2A). The ectopic PDGFRα expression markedly enhanced cell proliferation (Figure 2D up panel) and colony formation (Figure 2E up panel). Consistent results were obtained in Tsc1-deficient MEFs overexpressing PDGFRα (Figure 2B, 2D middle panel, and 2E middle panel). Moreover, depletion of PDGFRα using shRNAs targeting PDGFRα (shPDGFRα) in Tsc2+/+ MEFs inhibited cell proliferation and colony formation (Figure 2C, 2D low panel, and 2E low panel).

Next we investigated the role of PDGFRα in TSC tumors *in vivo*. Tsc2−/− MEFs transfected with lentiviral vector encoding PDGFRα or empty vector were subcutaneously injected into the right anterior armpit of nude mice, and then tumor growth was monitored. As depicted in Figure 2F, overexpression of PDGFRα dramatically enhanced the tumorigenic capacity of Tsc2−/− MEFs. IHC analysis revealed that tumor tissues derived from mice with injection of PDGFRα-overexpressing Tsc2−/− MEFs exhibited much stronger staining for Ki-67 than those in the control group (Figure 2H). Consistent results were obtained in mice with injection of PDGFRα-overexpressing Tsc1−/− MEFs and control mice (Figure 2G and 2I).

### PDGFRα is essential for AKT activation

**Figure 3:**
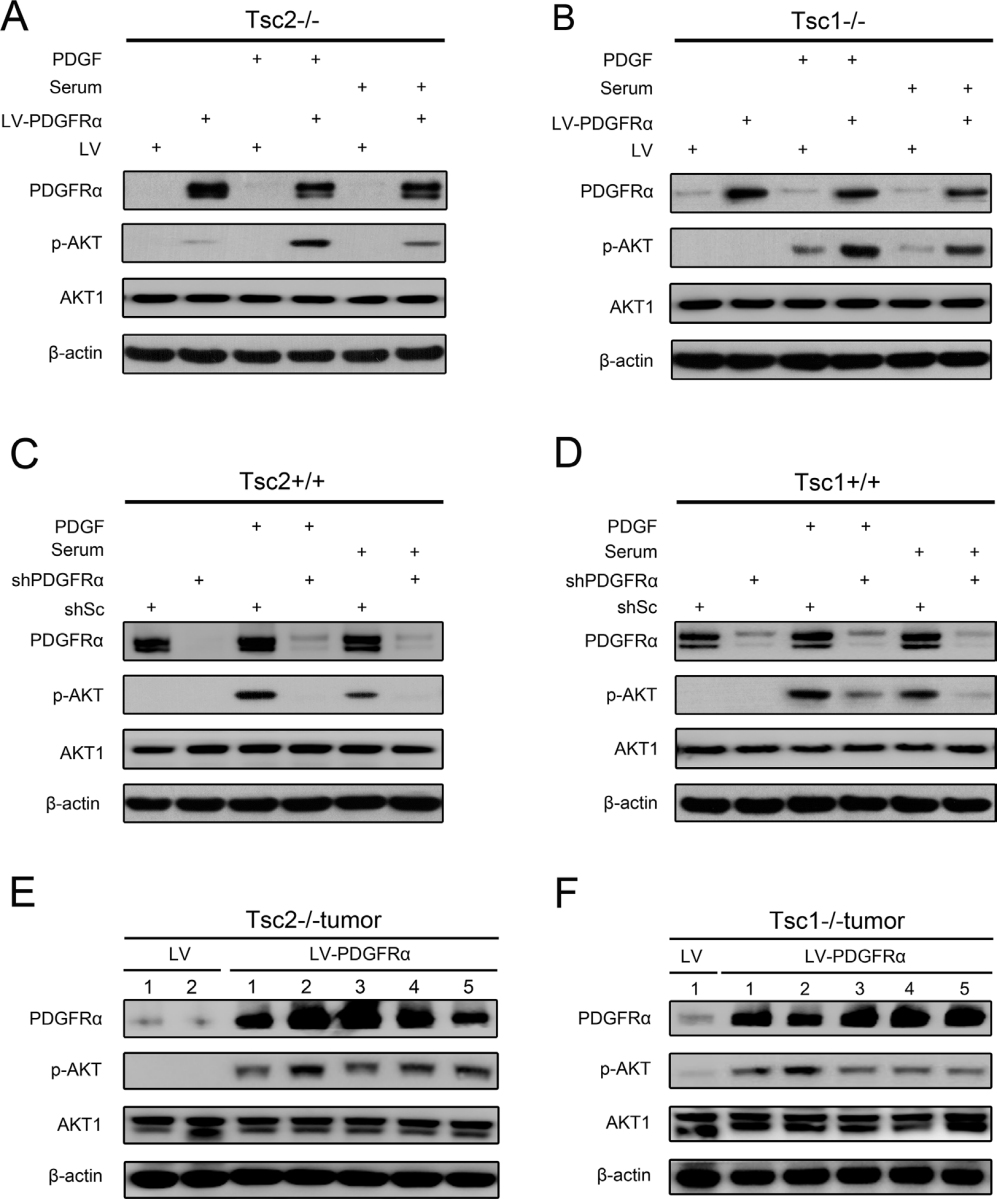
PDGFRα is essential for AKT activation (**A**, **B**) Tsc2−/− (A) or Tsc1−/− MEFs (B) were transduced with LV-PDGFRɑ or LV lentiviruses. (**C**, **D**) Tsc2+/+ (C) or Tsc1+/+ MEFs (D) were transduced with shPDGFRɑ or shSc lentiviruses. A–D. Cells were starved in DMEM for 24 h, followed by stimulation with serum or PDGF (50 ng/ml) for 30 min, and then cell lysates were harvested and subjected to immunoblotting with the indicated antibodies. (**E**, **F**) Tumor tissues derived from Tsc2−/− (E) or Tsc1−/− MEFs (F) transduced with LV-PDGFRɑ or LV lentiviruses were subjected to immunoblotting with the indicated antibodies.

Since the activation of mTORC1 led to downregulated expression of PDGFRα and inhibition of AKT in Tsc1- or Tsc2-null MEFs, thus we speculated that decreased PDGFRα is, at least in part, responsible for the attenuated AKT activity due to hyperactivation of mTORC1. To test this hypothesis, Tsc2−/− MEFs with overexpression of PDGFRα and the control cells were starved and then treated with fetal bovine serum (FBS) or PDGF for up to 30 min. As shown in Figure 3A, both serum and PDGF stimulation enhanced the activation of AKT induced by PDGFRα overexpression in Tsc2−/− MEFs. Similarly, PDGFRα overexpression also augmented serum or PDGF-stimulated AKT activation in Tsc1−/− MEFs (Figure 3B). Moreover, reduction of PDGFRα markedly attenuated AKT activation induced by serum or PDGF in Tsc2+/+ or Tsc1+/+ MEFs (Figure 3C and 3D). Furthermore, an elevated level of p-AKT was observed in tumor tissues derived from PDGFRα-overexpressing Tsc2−/− or Tsc1−/− MEFs as compared to the control tissues (Figure 3E and 3F). Collectively, these data reveal that PDGFRα is critical for AKT activation and loss of TSC1/TSC2 complex downregulates AKT activity partially through inhibition of PDGFRα expression.

### mTORC1 negatively regulates FOXO3a in Tsc1- or Tsc2-null MEFs

**Figure 4:**
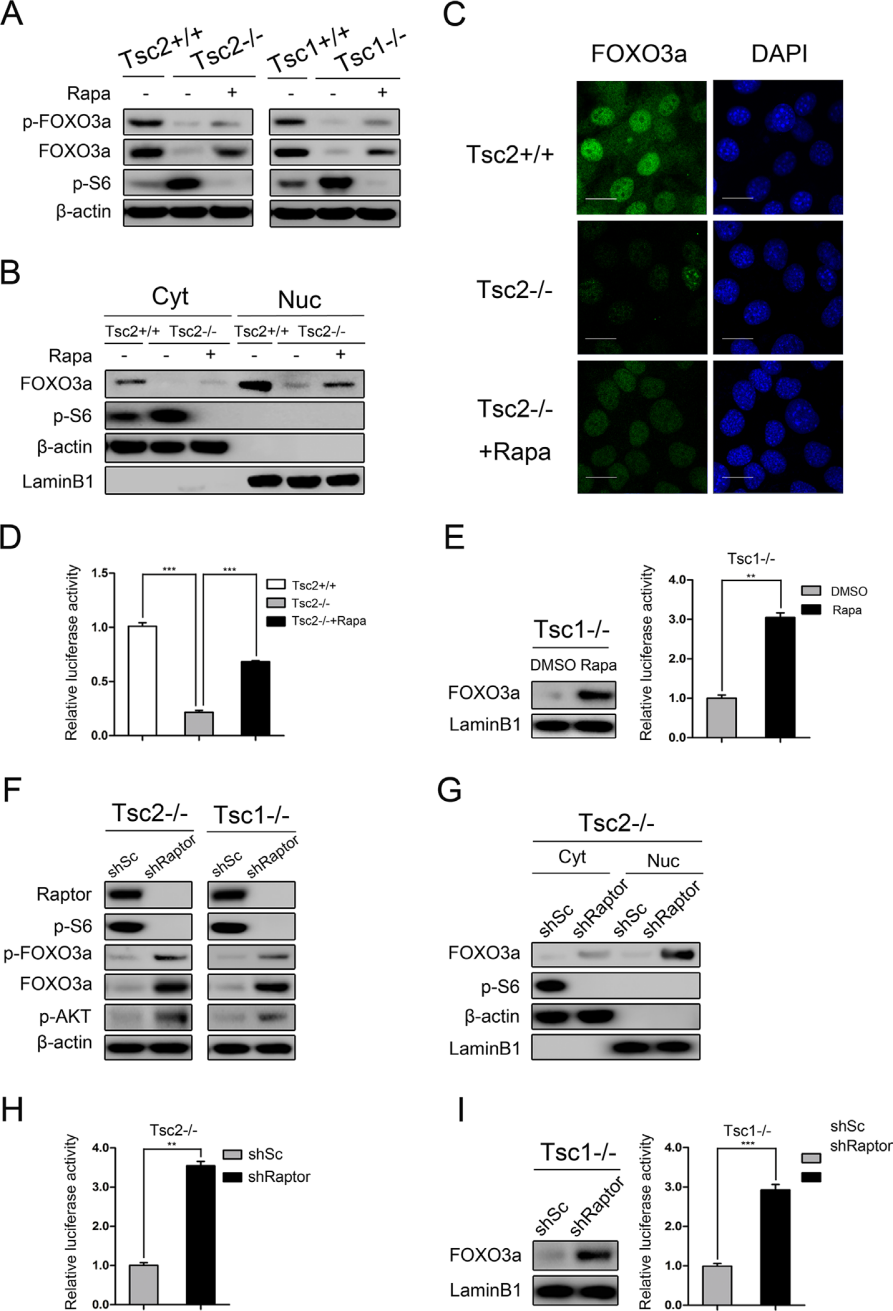
mTORC1 is a negative regulator of FOXO3a (**A**) Total cell lysates from Tsc2+/+, Tsc2−/−, rapamycin-treated (20 nM 24 h) Tsc2−/−, Tsc1+/+, Tsc1−/− and rapamycin-treated (20 nM 24 h) Tsc1−/− MEFs were subjected to immunoblotting with the indicated antibodies. (**B**–**D**) Tsc2+/+, Tsc2−/−, and rapamycin-treated (20 nM 24 h) Tsc2−/− MEFs. B. The cytoplasm (Cyt) and nuclear (Nuc) proteins were subjected to immunoblotting with the indicated antibodies. C. The expression of FOXO3a was analyzed by an immunofluorescence assay. Scale bar, 50 μm. D. The cells were co-transfected with pGMFOXO-Luc (200 ng) and the internal control plasmid pRL-TK (20 ng). The relative luciferase activity was measured 24 h after transfection. (**E**) Tsc1−/− MEFs were treated with or without rapamycin (20 nM) for 24 h. The nuclear proteins were subjected to immunoblotting with the indicated antibodies (left panel). The relative luciferase activity was measured as D (right panel). (**F**) Total cell lysates from Tsc2−/− or Tsc1−/− MEFs transduced with shRaptor or shSc lentiviruses were subjected to immunoblotting with the indicated antibodies. G-H. Tsc2−/− MEFs were transduced with shRaptor or shSc lentiviruses. (**G**) The cytoplasm (Cyt) and nuclear (Nuc) proteins were subjected to immunoblotting with the indicated antibodies. (**H**) The cells were co-transfected with pGMFOXO-Luc (200 ng) and the internal control plasmid pRL-TK (20 ng). The relative luciferase activity was measured 24 h after transfection. (**I**) Tsc1−/− MEFs were infected with shRaptor or shSc lentiviruses. The nuclear proteins were subjected to immunoblotting with the indicated antibodies (left panel). The relative luciferase activity was measured as H (right panel). **P < 0.01; ***P < 0.001.

The transcription factor FOXO3a is a positive regulator of cell apoptosis and cell cycle arrest and is frequently dysregulated in cancer [32, 33]. In many cancer cells, FOXO3a is phosphorylated and translocated to the cytoplasm due to aberrantly activated AKT, leading to inhibition of target gene transcription [32, 34]. As shown in Figure 4A, both p-FOXO3a (Ser253) and total FOXO3a were dramatically decreased in cells lacking TSC1/TSC2 complex as comparison with the control cells and were partially rescued by mTORC1 inhibition. Interestingly, nucleocytoplasmic separation experiments (Figure 4B) and immunofluorescence analysis (Figure 4C) showed that the nuclear level of FOXO3a was also dramatically reduced in Tsc2−/− MEFs and was markedly elevated by rapamycin treatment. These findings indicate that both the expression level and the nuclear accumulation of FOXO3a are negatively regulated by mTORC1, regardless of AKT activity. Furthermore, luciferase reporter assay confirmed that the transcriptional and DNA-binding activity of FOXO3a was significantly decreased in Tsc2-null MEFs and was rescued by rapamycin (Figure 4D). A consistent result was obtained in Tsc1−/− MEFs in response to rapamycin treatment (Figure 4E). In addition, we also determined whether FOXO3a is controlled by mTORC1 by using lentiviral vectors expressing shRNAs targeting Raptor in Tsc2−/− MEFs. As shown in Figure 4F, knockdown of Raptor strikingly decreased mTORC1 activity and increased the expression of p-AKT. Depletion of Raptor led to dramatically upregulated both total FOXO3a and nuclear FOXO3a, and significantly increased FOXO3a transcriptional activity (Figure 4F, 4G and 4H). Similarly, reduction of Raptor dramatically increased nuclear FOXO3a and its transcriptional activity in Tsc1−/− MEFs (Figure 4I). Taken together, both expression level and activity of FOXO3a were negatively regulated by mTORC1 in cells lacking TSC1/TSC2 complex.

### mTORC1 downregulates PDGFRα/AKT pathway through suppression of FOXO3a

**Figure 5:**
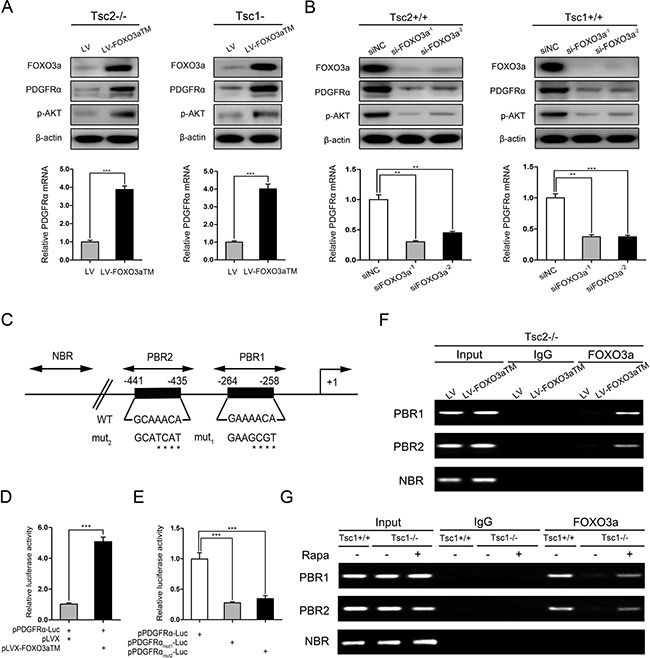
mTORC1 downregulates PDGFRɑ/AKT pathway through inhibition of FOXO3a (**A**) Tsc2−/− or Tsc1−/− MEFs were infected with lentivirus harboring a vector encoding FOXO3aTM (LV-FOXO3aTM) or the empty vector (LV). (**B**) Tsc2+/+ or Tsc1+/+ MEFs were transfected with two independent siRNAs targeting FOXO3a or the control siRNAs (siNC) for 48 h. A and B. Cell lysates and RNA were subjected to immunoblotting (upper panels) and qRT-PCR (lower panels), respectively. (**C**) Schematic representation of the putative wild-type (WT) and mutated (mut_1_ and mut_2_) FOXO3a-binding sites in the promoter of mouse PDGFRɑ gene. (**D**) HEK293T cells were co-transfected with pPDGFRɑ-Luc reporter plasmid plus FOXO3aTM expression vector (pLVX-FOXO3aTM) or control vector (pLVX) and the internal control plasmid pRL-TK. (**E**) Tsc2−/− MEFs were co-transfected with pLVX-FOXO3aTM plus pPDGFRɑ-Luc, pPDGFRɑ_mut1_-Luc, or pPDGFRɑ_mut2_-Luc reporter plasmid and pRL-TK plasmid. D and E. Relative luciferase activity was examined 24 h after transfection. (**F**) Tsc2−/− MEFs were transduced with LV-FOXO3aTM or LV lentiviruses. (**G**) Tsc1+/+, Tsc1−/−, or rapamycin-treated (20 nM 24 h) Tsc1−/− MEFs. F and G. Cells were subjected to ChIP assay using an anti-FOXO3a antibody. Normal rabbit IgG antibody served as the negative control. PCR was performed to amplify regions surrounding the putative FOXO3a-binding regions (PBR1 and PBR2) and a nonspecific FOXO3a-binding region (NBR). Representative data from two independent experiments were shown. **P < 0.01; ***P < 0.001.

Activation of mTORC1 led to both inhibition of FOXO3a and downregulated PDGFRα expression, so we next investigated whether mTORC1 downregulation of PDGFRα is mediated by FOXO3a. Tsc2- or Tsc1-null MEFs were transfected with lentiviruses overexpressing FOXO3aTM (a constitutively active FOXO3a). Ectopic expression of FOXO3aTM significantly promoted the expression of PDGFRα and p-AKT in Tsc2−/− or Tsc1−/− MEFs (Figure 5A upper panels). qRT-PCR analysis showed that FOXO3a upregulated the mRNA level of PDGFRα (Figure 5A lower panels). Conversely, depletion of FOXO3a with RNA interference decreased the levels of PDGFRα and p-AKT in Tsc2+/+ or Tsc1+/+ MEFs (Figure 5B). Collectively, mTORC1 downregulates PDGFRα/AKT signaling cascade via inhibition of FOXO3a.

To further elucidate the mechanisms underlying FOXO3a regulation of PDGFRα, we analyzed the 5′-flanking sequence of the PDGFRα gene upstream of the transcription start site. Two conserved FOXO3a binding sequences (−441/−435 GCAAACA; −264/−258 GAAAACA) were identified within the promoter of the mouse PDGFRα gene (Figure 5C). We then cloned the mouse PDGFRα gene promoter (−582/+68) into a luciferase reporter vector and evaluated the effect of FOXO3a on the promoter activity. Luciferase reporter assay in HEK293T cells showed that overexpression of FOXO3aTM increased luciferase activity (Figure 5D), indicating that FOXO3a directly regulates PDGFRα transcription. Moreover, the enhanced transcriptional activity was attenuated when either of the two putative FOXO3a-binding sequences were mutated (Figure 5E). ChIP analysis further revealed that FOXO3a bound to these two putative sites (Figure 5F). In addition, the direct binding of FOXO3a to the two putative sites within the PDGFRα gene promoter was drastically weaker in Tsc1−/− MEFs than in the control cells, and was rescued by suppression of mTORC1 with rapamycin (Figure 5G). Taken together, FOXO3a promotes the transcription of PDGFRα through directly binding to the promoter of PDGFRα gene.

### Rapamycin in combination with AG1295 inhibits the growth of cells lacking TSC1/TSC2 complex *in vitro* and *in vivo*

**Figure 6:**
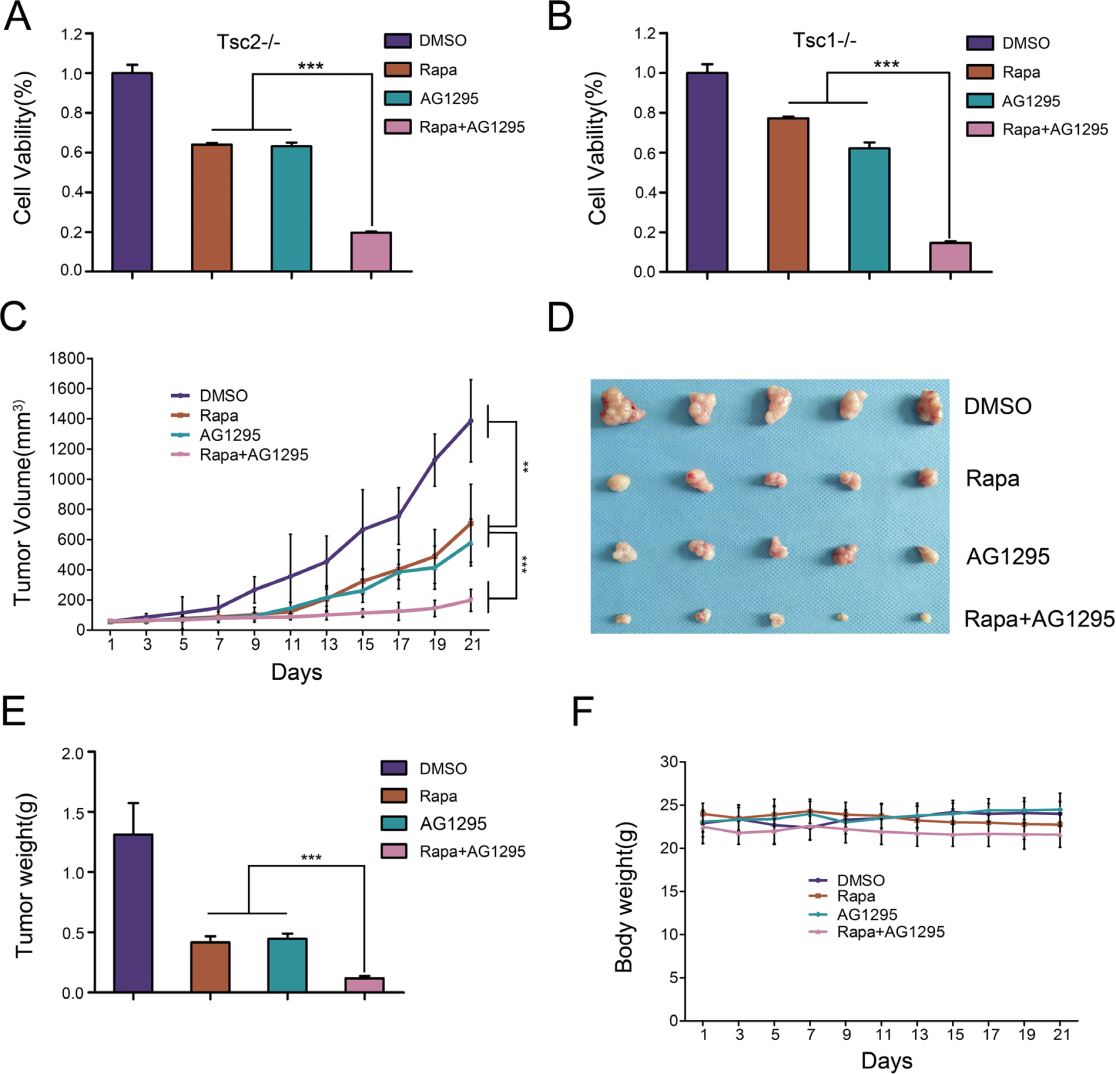
The combination of rapamycin and AG1295 more effectively suppresses the growth of cells lacking TSC1/TSC2 complex *in vitro* and *in vivo* than either agent alone (**A**, **B**) Tsc2−/− (A) or Tsc1−/− MEFs (B) were treated with a combination of 5 nM rapamycin (Rapa) and 20 μM AG1295 or either agent alone for 48 h. Cell viability was examined with an MTT assay. (**C**–**F**) NTC/T2-null cells were inoculated subcutaneously into the nude mice to evaluate the effects of rapamycin and AG1295 *in vivo*. (C) Tumor volume growth curves. (D) Dissected tumors. (E) Tumor weight. (F) Body weights of mice. **P < 0.01; ***P < 0.001.

Since the inhibition of mTORC1 markedly increased PDGFRα expression and then led to activation of AKT, we next evaluated whether the combination of rapamycin with AG1295 (a specific PDGFR inhibitor) could achieve a better inhibitory effect on the growth of cells lacking TSC1/TSC2 complex. As depicted in Figure 6A, the combination treatment of rapamycin and AG1295 exerted a stronger inhibitory effect on cell viability of Tsc2−/− MEFs than either compound alone, which was consistent with the result in Tsc1−/− MEFs (Figure 6B). Next we explored the *in vivo* effect of combination administration of rapamycin and AG1295 by using a mouse tumor model with NTC/T2-null cells (a cell line with potent tumorigenicity derived from Tsc2−/− MEFs) [28]. As shown in Figure 6C–6E, the tumor volumes and tumor weights were significantly inhibited by treatment with rapamycin or AG1295 alone, and rapamycin in combination with AG1295 exerted a better anti-tumor activity than the treatment with either drug alone. Moreover, the combination treatment did not significantly affect the body weights of the mice (Figure 6F). Therefore, AG1295 enhances the inhibitory effect of rapamycin on the tumorigenic capacity of cells lacking TSC1/TSC2 complex.

## DISCUSSION

Overactivation of AKT is a hallmark of many human tumors [35]. Although TSC is a genetic disease with tumors formation in multiple organs [9], AKT activity is reduced in TSC tumor cells [17–20]. This phenomenon contributes to explain why most TSC patients are inclined to develop benign tumors [9, 36–38]. However, the precise mechanisms underlying the reduced AKT activity due to loss of TSC1/TSC2 complex remain incompletely understood. It has been suggested that mTORC1-mediated regulation of several molecules, such as Grb10, IRS, and PTEN, are involved in the suppression of AKT activity in TSC cells [18, 20, 39]. Recently, we have also identified an mTORC1-independent manner which mediates AKT inhibition in TSC lesions [40]. Here we showed that loss of TSC1/TSC2 complex downregulated PDGFRα expression via activation of mTORC1 (Figure 1). Ectopic expression of PDGFRα enhanced serum- or PDGF-stimulated AKT activation in Tsc1- or Tsc2-null MEF cells (Figure 3). Moreover, overexpression of PDGFRα accelerated cell proliferation and tumor growth of Tsc1- or Tsc2-null MEFs (Figure 2). Thus, mTORC1 suppresses AKT activity through downregulation of PDGFRα, which ameliorates the malignancy of TSC.

Although mTORC1 is recognized as the pivotal regulator of protein translation, accumulating evidence shows that mTORC1 affects gene expression through regulation of transcription factors, such as STAT3, HIF1α, and etc. [20, 27, 28, 30, 41]. In this study, we demonstrated that the transcription factor FOXO3a, as a downstream target of mTORC1, transcriptionally regulates PDGFRα expression in Tsc1- or Tsc2-deficient cells. This findings not only confirmed a previous study which showed that FOXO3a transcriptionally regulates the expression of PDGFRα in neuroblastoma cells [42], but also revealed that FOXO3a activity is negatively regulated by mTORC1 (Figure 4), which was consistent with a previous study indicating that inactivation of mTORC1 induces nuclear accumulation of FOXO3a [43]. In addition to suppression of FOXO3a transcriptional activity, mTORC1 inhibited the expression of FOXO3a at both protein and mRNA levels (Figure 4 and Supplementary Figure 2). It has been reported that the expression of FOXO3a could be epigenetically regulated [43]. However, treatment with 5-aza-deoxycytidine (5-aza-dC) or trichostatin A (TSA) had a minimal effect on the expression of FOXO3a in Tsc2−/− MEFs (Supplementary Figure 3), indicating that the epigenetic regulation was not implicated in mTORC1-mediated downregulation of FOXO3a in cells lacking TSC1/TSC2 complex. In addition to mTORC1, mTORC2 has also been reported to be involved in the regulation of FOXO3a [44–46]. Intriguingly, herein we found that inhibition of mTORC1, but not mTORC2, upregulated the expression level and activity of FOXO3a in Tsc1- or Tsc2-deficient MEFs (Figure 4 and Supplementary Figure 4). This discrepancy could be explained by the findings that mTORC2 activity has been inhibited by loss of TSC1/TSC2 complex [47, 48], and therefore knockdown of Rictor, a specific component of mTORC2, can no longer reduce mTORC2 activity as well as its downstream targets in Tsc1- or Tsc2-null MEFs.

It is well established that AKT phosphorylation of FOXO3a leads to FOXO3a sequestration in the cytoplasm and inactivates its transcription activities [34]. Interestingly, emerging evidence indicates that FOXO3a can activate AKT. Chen etc. found that constitutively nuclear localization of FOXO3a promotes AKT phosphorylation in breast cancer [49]. Hui and colleagues reported that FOXO3a upregulates AKT activity through induction of PI3KCA expression in drug-resistant leukemic cells [50]. In addition, FOXO3a has been also reported to enhance the expression of Rictor, leading to increased AKT phosphorylation [51, 52]. In line with these previous studies, we found that FOXO3a positively regulates AKT activity in WT cells or Tsc1- or Tsc2-deficient cells (Figure 5). Moreover, PDGFRα was identified as a direct downstream target of FOXO3a, which mediates the activation of AKT (Figure 3 and Figure 5). In addition, we displayed that aberrantly activated mTORC1 inhibits FOXO3a, leading to downregulated expression of PDGFRα and consequently decreased AKT activity. Thus, downregulated FOXO3a/PDGFRα/AKT pathway may serve as a protective mechanism against TSC-associated tumor development.

Since aberrant activation of mTORC1 is considered to be mainly responsible for TSC-related tumor formation, rapamycin and its analogues have been suggested to be effective drugs for TSC treatment [9, 53–55]. However, the therapeutic efficacy of such drugs is limited by several reasons, including immunosuppression-associated opportunistic infections [56] and reactivation of AKT [17, 18]. Here we demonstrated that rapamycin treatment upregulated PDGFRα expression (Figure 1), and overexpression of PDGFRα accelerated cell proliferation and tumorigenesis of Tsc1- or Tsc2-null MEFs (Figure 2). It is a widely used strategy for tumor treatment to reduce cytotoxicity and increase treatment efficacy by rational combination of different drugs. So we hypothesized that combined administration of rapamycin and a PDGFR inhibitor would achieve a better therapeutic effect than rapamycin alone in TSC treatment. As shown in Figure 6, combination of rapamycin and AG1295 exhibited a stronger inhibitory effect on the growth of cells lacking TSC1/TSC2 complex *in vitro* and *in vivo*. Therefore, co-administration of rapamycin and a PDGFR inhibitor may be an effective and novel strategy for the treatment of TSC.

**Figure 7:**
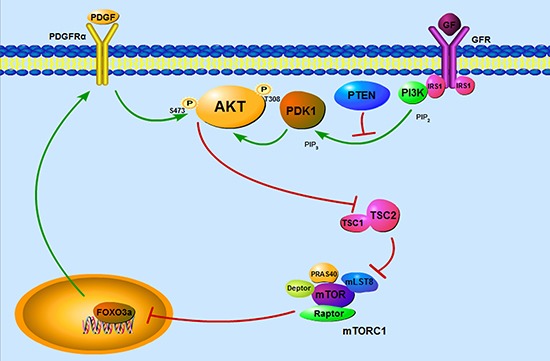
Schematic illustration of TSC1/TSC2/mTORC1 pathway regulation of PDGFRɑ/AKT signaling cassette through FOXO3a Hyperactivation of mTORC1 induced by loss of TSC1/TSC2 complex results in feedback inhibition of AKT signaling through downregulation of FOXO3a/PDGFRɑ cascade, which contributes to the benign nature of TSC-associated tumors.

In conclusion, we have demonstrated that mTORC1 activation due to loss of TSC1/TSC2 complex led to feedback inhibition of AKT signaling through downregulation of FOXO3a/PDGFRɑ cascade (Figure 7). This study shed new light on the underlying mechanism of benign tumor formation in TSC patients and contributes to a better understand of mechanisms involved in FOXO3a-mediated activation of AKT. Importantly, the results of our work suggest that combined administration of rapamycin with a PDGFR inhibitor may be a novel strategy for the treatment of TSC-related tumors.

## MATERIALS AND METHODS

### Reagents, plasmids, and antibodies

Rapamycin was obtained from Selleck Chemicals (Houston, TX, USA). Tyrphostin AG1295 was from Santa Cruz Technology (Santa Cruz, CA, USA). 5-aza-deoxycytidine (5-aza-dC) and trichostatin A (TSA) were from Sigma-Aldrich (St Louis, MO, USA). Murine PDGF-AA was acquired from Pepro Tech (Rocky Hill, NJ, USA). Fetal bovine serum (FBS), Dulbecco's Modified Eagle's Medium (DMEM), Lipofectamine 3000, and NuPAGE 4–12% Bis-Tris Gels were purchased from Life Technologies (Carlsbad, CA, USA). pLVX-IRES-Puro vector was from Clontech (Mountain View, CA, USA). The FOXO luciferase reporter plasmid pGMFOXO-Luc was purchased from Genomeditech (Shanghai, China). A constitutively active FOXO3a (FOXO3aTM), in which all three AKT phosphorylation sites were mutated to alanine, was obtained from Addgene (Cambridge, MA, USA). Antibodies against TSC1, Raptor, Rictor, phospho-S6 (Ser235/236), PDGFRɑ, FOXO3a, AKT1 and phospho-AKT (Ser473) were from Cell Signaling Technology (Danvers, MA, USA). LaminB1, phospho-FOXO3a (Ser253) and Ki-67 antibodies were from Abcam (Cambridge, MA, USA). TSC2, β-actin, and all HRP-labeled secondary antibodies were from Santa Cruz Technology.

### Cell culture

Tsc2+/+, Tsc2−/−, Tsc1+/+, Tsc1−/− MEFs, and NTC/T2-null cells have been described previously [17, 27, 28]. HEK293T cells were obtained from the ATCC (Manassas, VA, USA). All cells were maintained and propagated in DMEM with 10% FBS and 1% penicillin/streptomycin in 5% CO_2_ at 37°C.

### Cell fractionation and immunoblotting

Cytoplasmic and nuclear protein fractions were extracted with a NE-PER Reagent Kit (Pierce, Rockford, IL, USA) according to the manufacturer's instructions. Cell or tumor tissue lysates were separated by NuPAGE 4–12% Bis-Tris Gels (Life Technologies), and then transferred to PVDF membrane (Millipore, Billerica, MA, USA), followed by incubation with appropriate primary antibodies and horseradish peroxidase (HRP)-conjugated secondary antibodies. Immunoreactive bands were visualized with Pierce^™^ ECL Western Blotting Substrate (Thermo Scientific, Waltham, MA, USA) according to the manufacturer's protocol.

### Quantitative real time-PCR (qRT-PCR)

Total RNA was isolated from cells using TRIzol reagent (Life Technologies) according to the manufacturer's instructions. 1 μg of RNA was converted into cDNA using the RevertAid™ First Stand cDNA Synthesis Kit (Fermentas, Waltham, MA, USA). After 10-fold dilution, 4 μl of cDNA was subjected to PCR amplification using SYBR Premix Ex Taq^TM^ II (TaKaRa, Shiga, Japan) according to the manufacturer's protocol in a StepOnePlus^™^ Real-Time PCR System (ABI, Foster City, CA, USA). β-actin served as the internal control. The primer sequences were as follows: PDGFRɑ forward, 5′-ACACGTTTGAGCTGTCAACC-3′, and reverse, 5′-CCCGACCACACAAGAACAGG-3′; FOXO3a forward, 5′-CTGGGGGAACCTGTCCTATG-3′, and reverse, 5′-TCATTCTGAACGCGCATGAAG-3′; β-actin forward, 5′-AGAAAATCTGGCACCACACC-3′, and reverse, 5′-AGAGGCGTACAGGGATAGCA-3′.

### RNA interference

Cells were seeded into 12-well plates and transfected with siRNAs using siRNA-Mate (GenePharma, Shanghai, China) according to the manufacturer's protocol. All siRNA oligonucleotides were synthesized by GenePharma. The siRNA target sequences were as follows: Raptor, 5′-GGACAACGGTCACAAGTAC-3′; FOXO3a^−1^, 5′-CAT GCGCGTTCAGAATGAA-3′; FOXO3a^−2^, 5′-GAACG TTGTTGGTTTGAAT-3′; Negative Control (NC), 5′-TTC TCCGAACGTGTCACGT-3′.

### Preparation of recombinant plasmids and viruses

GV248 lentiviral shRNA expression vectors targeting mouse PDGFRɑ, mouse Raptor, mouse Rictor, and the control scrambled shRNA (shSc) were obtained from Genechem (Shanghai, China). The target sequence for PDGFRɑ was 5′-CCTGGAGAAGTGAGAAACAAA-3′. The target sequence for Rictor was 5′-GCCCTA CAGCCTTCATTTA-3′. The target sequence for Raptor is consistent with the sequences used in RNA Interference as above. GV367 lentiviral plasmid expressing mouse PDGFRɑ and the empty plasmid were obtained from Genechem (Shanghai, China). The FOXO3aTM expression vector was constructed by subcloning FOXO3aTM cDNA (Addgene) into the pLVX-IRES-Puro vector. All recombinant plasmids were verified by DNA sequencing. The recombinant plasmids and empty control vector were named as LV-PDGFRɑ, LV-FOXO3aTM, and LV, respectively. Lentiviruses were generated by co-transfecting a recombinant vector or the corresponding control vector with the packaging vectors (pVSVG, pREV, and pMDL) into HEK293T cells. After 48 h of transfection, culture supernatants were collected and filtered with a 0.45 μm filter, and then used to infect target cells as described previously [27].

### Cell proliferation and viability assay

Cell proliferation was measured by an MTT assay according to the manufacturer's instructions and has been described previously [29]. For the cell viability assays, Tsc1- or Tsc2-null MEFs were seeded in 96-well plates at a density of 3, 000 cells/well and treated with DMSO, rapamycin, AG1295, or combination of rapamycin and AG1295 for 48 h. Followed by added 10 μl of Cell Counting Kit-8 reagent (Beyotime, China) to per well and incubated the plates for 1 h, the optical density (OD value) of each well was measured at 450 nm.

### Colony formation assay

Cells were seeded into 100 mm cell culture dish at a density of 200 cells per dish. After incubation for approximately 2 weeks in DMEM containing 10% FBS, the cells were fixed and dyed with 0.1% crystal violet (1 mg/ml). The number of colonies containing over 50 cells was counted.

### Immunofluorescence assay

FOXO3a was analyzed by immunofluorescence assay. Briefly, cells were seeded on coverslips overnight and then fixed with 4% formaldehyde for 5 min at room temperature, and followed by the treatment of 1% Triton X-100 (Sigma-Aldrich) for permeabilization. After blocking with 2% bovine serum albumin for 1 h, the cells were incubated with anti-FOXO3a antibody (Cell Signaling Technology) for overnight and then incubated with FITC-conjugated secondary antibody (Cell Signaling Technology) for 1 h. DAPI (Sigma-Aldrich) was used to visualize cell nucleus and the fluorescence staining was examined under a FV1000 confocal microscope (Olympus, Tokyo, Japan).

### Induction of subcutaneous tumors and combination treatment of rapamycin and AG1295

Immunodeficient BALB/c nude mice (5 weeks old) were purchased from Vital River Laboratory Animal Technology (Beijing, China). 3 × 10^6^ cells as the indicated in 0.2 ml DMEM were inoculated subcutaneously into the right anterior armpit of mice and the tumor growth was monitored. Five mice were used in each cohort. The mice were euthanized and imaged on day 50 after inoculation. Subcutaneous tumors were established as described previously [18, 30].

To evaluate the therapeutic efficacy of rapamycin and AG1295 *in vivo*, NTC/T2-null cells (3 × 10^6^ cells) were injected subcutaneously near the axillary fossa of nude mice. The mice were randomly divided into four groups (five mice per group) when the tumor volumes reached 50–70 mm^3^. And then the mice in four different groups were treated with vehicle solution (75% DMSO and 25% PBS), rapamycin (2 mg/kg), AG1295 (5 mg/kg), or combination of rapamycin (2 mg/kg) and AG1295 (5 mg/kg), respectively (i.p., every two days, a total of ten injections). Tumor dimensions were measured with a digital caliper every two days, and the tumor volume was calculated using the formula V = 1/2 (width^2^ × length). Body weights were also monitored. On day 34 after inoculation of tumor cells, all experimental mice were terminated with ether anesthesia and the total weight of tumors in mice were measured. All animals were maintained and used in accordance with the guidelines of the Animal Center of Anhui Medical University, and all animal experimental procedures were approved by the Experimental Animal Ethical Committee of Anhui Medical University.

### Immunohistochemistry (IHC)

Immunohistochemical analysis was performed as described previously [31]. In brief, tumor tissues were fixed in 4% paraformaldehyde and embedded in paraffin. Sections (4 μm) were prepared for the indicated primary antibodies or hematoxylin and eosin (H&E) staining according to standard protocols.

### Promoter-reporter constructs and luciferase reporter assay

A 650-bp fragment of the mouse PDGFRɑ promoter (−582/+68) was obtained by PCR using mouse genomic DNA extracted from Tsc2+/+ MEFs. Primer sequences used were as follows: forward, 5′-TTGAAATGCGTGCAAACGCTGAGCATAG-3′; reverse, 5′-CTGCGACCTGAGAGGAGACTGGGCA-3′. The amplified DNA fragments were cloned into the pGL3-Basic (Promega, Madison, WI, USA) plasmid through the *Kpn* I and *Xho* III sites. The potential FOXO3a-binding sites on the promoter of mouse PDGFRɑ gene were mutated using the Q5 Hot Start High-Fidelity DNA Polymerase (NEB, Ipswich, MA, USA). The primer sequences were as follows: PDGFRɑ_mut1_, forward, 5′-AGGGGCAGGGCATCATAAGGGGCAAGGT-3′, reverse, 5′-GGGCACCCCTGCTCTACTTCATGCCT CTA-3′; PDGFRɑ_mut2_, forward, 5′-TCGCAGTTGAAGC GTATGCAAACGCTGA-3′, reverse, 5′-CCTGAGAGG AGACTGGGCAGGGTGGA-3′. For Luciferase reporter assays, cells were cultured in triplicate to 80% confluence in 24-well plates and co-transfected with the promoter constructs (200 ng) and the internal control plasmid pRL-TK (20 ng). Luciferase activity was detected with the Dual-Luciferase Reporter Assay System (Promega).

### Chromatin immunoprecipitation (ChIP)

ChIP assay with an anti-FOXO3a antibody (Abcam) to detect protein-DNA interactions were performed using a SimpleChIP^®^ Plus Enzymatic Chromatin IP kit (Cell Signaling Technology) according to manufacturer's protocol. The purified DNA was analyzed by standard PCR with Hot Start Taq DNA Polymerase (NEB). The primer sequences were as follows: the putative FOXO3a-binding region 1 (PBR1), forward, 5′-TGTGGCTCGCCTGTAACCCTAAAC-3′, reverse, 5′-ATACCAGGCGACACGGATCTACCC-3′; the putative FOXO3a-binding region 2 (PBR2), forward, 5′-GGGGATAAATGTAAGCCACGGACT-3′, reverse, 5′-CTGGACCAAGCTCTGTGATTCGG-3′; a nonspecific FOXO3a-binding region (NBR), forward, 5′-GGAAGTGTTGGCCTGTTTGTCTGC-3′, reverse, 5′-TCTGGGCAGCTCTGTCCGACG-3′.

### Statistics

All quantitative data were represented as mean ± SEM of at least three independent experiments. The difference between two groups was evaluated with 2-tailed Student's *t*-test. One-way ANOVA and Tukey post hoc test were used to evaluate differences of multiple comparisons. All statistical analyses were conducted using GraphPad Prism software. Differences were considered significant when *p* < 0.05.

## SUPPLEMENTARY MATERIALS FIGURES



## Abbreviations

TSC: tuberous sclerosis complex
mTOR: mammalian target of rapamycin
mTORC1: mammalian target of rapamycin complex 1
mTORC2: mammalian target of rapamycin complex 2
PDGFRα: platelet-derived growth factor receptor α
FOXO3a: forkhead box O3a
MEFs: mouse embryonic fibroblasts.
