# Supplementary material for: Hyperactivated mTORC1 downregulation of FOXO3a/PDGFRα/AKT cascade restrains tuberous sclerosis complex-associated tumor development
Source: Oncotarget. 2017 Jul 4;8(33):54858–72. doi: 10.18632/oncotarget.18963 (PMC5589626; doi:10.18632/oncotarget.18963)
Supplement: Supplementary file 1 [file oncotarget-08-54858-s001.pdf]

Hyperactivated mTORC1 downregulation of FOXO3a/PDGFRα/AKT cascade restrains tuberous sclerosis complex-associated tumor development

SUPPLEMENTARY MATERIALS

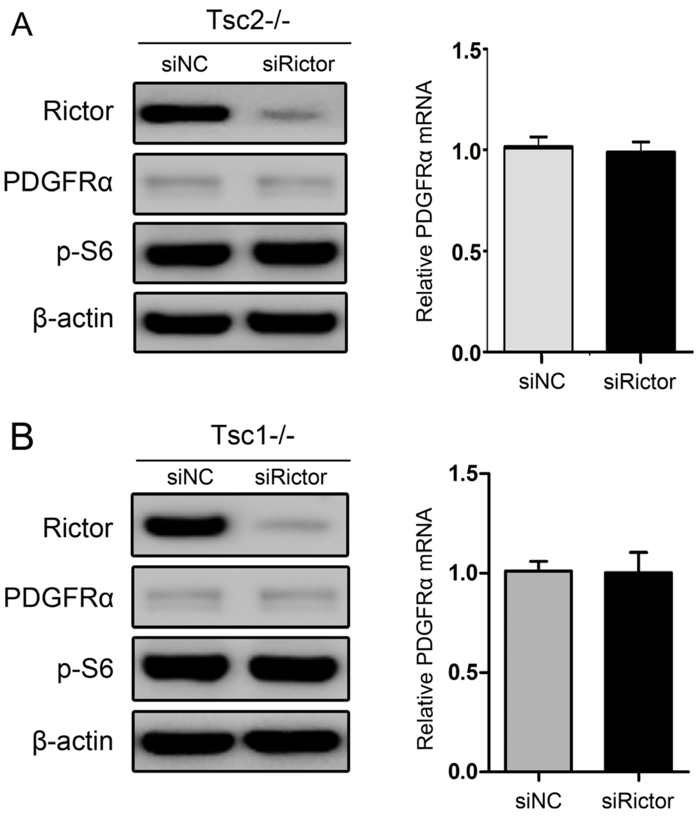

Supplementary Figure 1: mTORC2 has a little effect on the expression of PDGFRα in Tsc2- or Tsc1-null MEFs. (**A**, **B**) Tsc2-/- (A) or Tsc1-/- (B) MEFs were transfected with control siRNA (siNC) or siRNA for Rictor for 48 h. Cell lysates were subjected to immunoblotting (left panels); qRT-PCR was performed to analyze the level of PDGFRα mRNA (right panels).

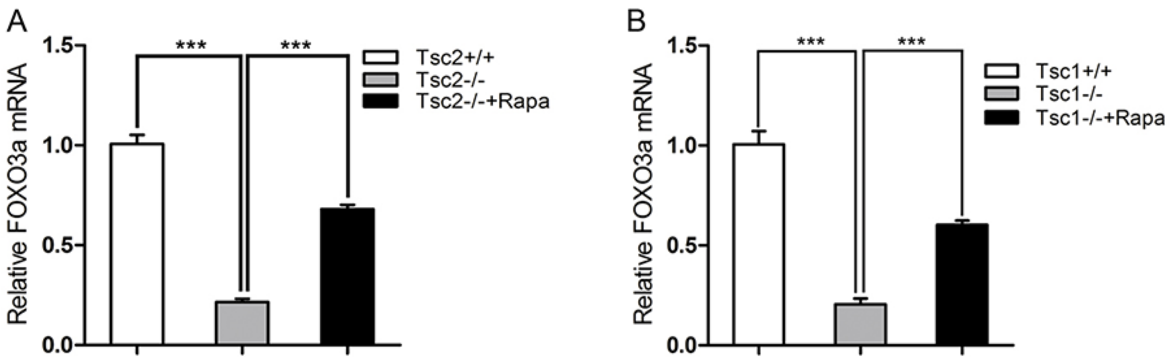

Supplementary Figure 2: Loss of TSC1 or TSC2 reduced the expression of FOXO3a mRNA through activation of mTORC1. (**A**) Tsc2+/+, Tsc2-/-, or rapamycin-treated (20 nM 24 h) Tsc2-/- MEFs. (**B**) Tsc1+/+, Tsc1-/-, or rapamycin-treated (20 nM 24 h) Tsc1-/- MEFs. A and B. qRT-PCR was performed to analyze the level of FOXO3a mRNA. ***$P < 0.001$.

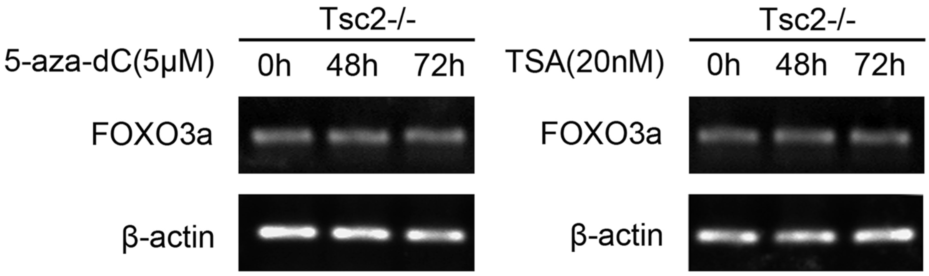

Supplementary Figure 3: Treatment with 5-aza-dC or TSA had a minimal effect on the expression of FOXO3a in Tsc2-null MEFs. Tsc2-/- MEFs were treated with 5 μM 5-aza-deoxycytidine (5-aza-dC) or 20 nM trichostatin A (TSA) for different times as indicated. RT-PCR was performed to analyze the mRNA level of FOXO3a.

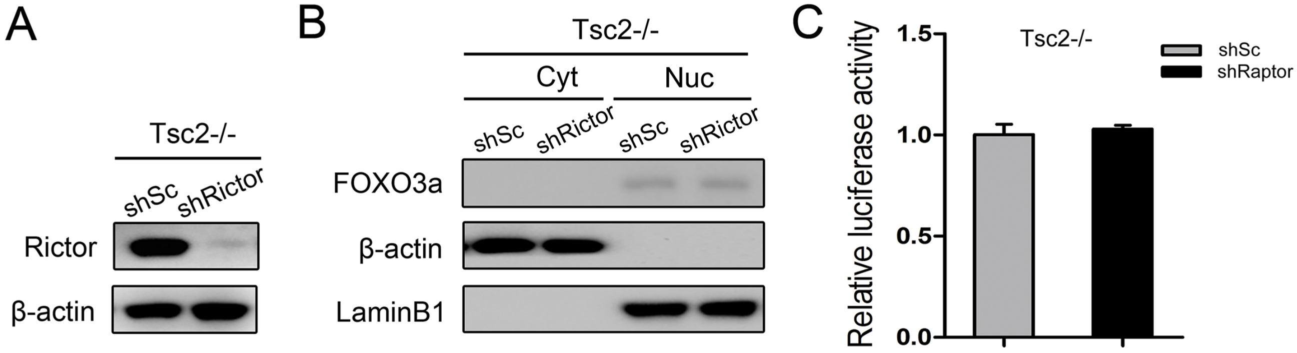

Supplementary Figure 4: Knockdown of Rictor has a little impact on the expression and activity of FOXO3a in Tsc2−/− MEFs. Tsc2-/- MEFs were infected with shRictor or shSc lentiviruses. (**A**, **B**) Total cell lysates (A) or the cytoplasm (Cyt) and nuclear (Nuc) proteins (B) were subjected to immunoblotting with the indicated antibodies. (**C**) The cells were co-transfected with pGMFOXO-Luc (200 ng) and the internal control plasmid pRL-TK (20 ng). The relative luciferase activity was measured 24 h after transfection.
